# **Anti-obesity effect of collagen peptides obtained from *Diplulmaris antarctica***, **a jellyfish of the Antarctic region**

**DOI:** 10.3325/cmj.2023.64.21

**Published:** 2023-02

**Authors:** Nataliia Raksha, Tetiana Halenova, Tetiana Vovk, Olexandra Kostyuk, Tatyana Synelnyk, Tatyana Andriichuk, Tetiana Maievska, Olexiy Savchuk, Ludmila Ostapchenko

**Affiliations:** 1 Department of Biochemistry, Educational and Scientific Center “Institute of Biology and Medicine,” Taras Shevchenko National University of Kyiv, Kyiv, Ukraine; 2 Ukrainian Fishing Industry Cluster of Innovations, Kyiv, Ukraine

## Abstract

**Aim:**

To investigate the ability of collagen peptides derived from a jellyfish of the Antarctic region (*Diplulmaris antarctica*) to prevent the development of obesity in rats fed a high-calorie diet.

**Methods:**

Collagen peptides were produced by pepsin hydrolysis of jellyfish-derived collagen. The purity of collagen and collagen peptides was confirmed by SDS-polyacrylamide gel electrophoresis. Rats were fed a high-calorie diet for ten weeks and were simultaneously orally administered collagen peptides (1 g per 1 kg of body weight every other day) starting from the fourth week. Body mass index (BMI), body weight gain, selected nutritional parameters, the key parameters associated with insulin resistance, and the level of oxidative stress markers were assessed.

**Results:**

Compared with untreated obese rats, rats treated with hydrolyzed jellyfish collagen peptides had a decreased body weight gain and body mass index. They also had a decreased level of fasting blood glucose, glycated hemoglobin, insulin, lipid peroxidation products (conjugated dienes, Schiff bases), and oxidatively modified proteins, as well as a restored activity of superoxide dismutase.

**Conclusion** Collagen peptides obtained from *Diplulmaris antarctica* can be used to prevent and treat obesity caused by a high-calorie diet and pathologies associated with increased oxidative stress. Given the obtained results and the abundance of *Diplulmaris antarctica* in the Antarctic region, this species can be considered a sustainable source of collagen and its derivatives.

Obesity increases the risk of many health disorders, including cardiovascular disease, kidney disease, diabetes mellitus, hypertension, and cancer (1-3). Although the existing medications for obesity treatment (4,5) are effective, their safety and side effects remain a concern. Therefore, there is a need to find natural substances to manage obesity with minimal side effects.

Peptides are currently being actively studied as possible alternatives to drugs for the prevention and treatment of various obesity complications. Endogenous and bioactive endogenous peptides possess a wide range of anti-oxidant, anti-inflammatory, anti-microbial, anti-ulcer, lipid-lowering, wound healing, and anti-skin-aging effects (6-8). In addition, peptides are a part of a complex system of energy balance regulation, which, when impaired, is one of the causes of obesity. Endogenous orexigenic and anorexigenic peptides responsible for regulating the onset of hunger, satiety, adipocyte metabolism, and inflammation have been identified (9,10).

A promising source for obtaining peptides is collagen. Collagen and its fragments are included in pharmaceutical preparations due to their excellent biocompatibility, biodegradability, and low antigenicity. Collagen peptides affect glucose tolerance and insulin sensitivity in overweight individuals, modulate the immune status, and have a hypocholesterolemic effect (11-13). Although sources of collagen are available and often quite cheap, frequent outbreaks of infectious diseases among land animals necessitate the search for alternative sources of proteins. Additionally, the use of molecules from warm-blooded animals may be unacceptable to individual patients due to their religious beliefs or lifestyle. In this context, hydrobionts, which make up about half of the world's biodiversity, can be an inexhaustible source of collagen. The rapid growth of the jellyfish population around the world seriously affects ecosystems and human activities in marine areas. On the other hand, for hundreds of years jellyfish has been an important food source in many countries (14). There is growing interest in biologically active compounds isolated from jellyfish, which were found to exhibit antioxidant (15), anti-microbial (16), anti-cancer (17), as well as immune-modulatory and wound healing properties (18). Jellyfish is rich in collagenous protein, which makes this species a promising source of collagen and collagen-related products. Despite the conservative structure, the collagen from hydrobionts has a lower content of alanine, glycine, and proline and a higher content of arginine, aspartic acid, threonine, tyrosine, cysteine, and methionine than the collagen isolated from mammals (19,20). Proteins isolated from organisms living in atypical habitats (low or high temperatures, high pressure, low illumination) may have certain structural and functional features (conformational flexibility, high catalytic efficiency, thermolability, or thermostability) (21). This allows us to suggest that the hydrobionts of the Antarctic region may contain compounds with new or more pronounced properties. *Diplulmaris antarctica* is abundant in the Antarctic region and may represent a sustainable source of collagen. Although the effects of collagen or total protein hydrolysates from jellyfish have already been investigated, the research using collagen peptides (less than 5 kDa) remains limited. In the current study, we evaluated the efficacy of collagen peptides obtained from *Diplulmaris antarctica* in preventing diet-induced obesity.

## Material and methods

### Preparation of collagen peptides

The jellyfish was caught near the island of Galindez (65°15′ S, 64°15′ W) in the Argentine Islands archipelago by the Ukrainian Antarctic expeditions. The whole jellyfish specimens were individually frozen in liquid nitrogen and stored at -80 °C to prevent enzyme deterioration. The samples were transported to the laboratory frozen. The jellyfish was authenticated by the Department of Zoology and Ecology of Educational and Scientific Center “Institute of Biology and Medicine” of Taras Shevchenko National University of Kyiv, Ukraine.

The jellyfish samples were washed at least three times with tap water and once with ultra-purified water. The clean jellyfish mass was homogenized with a blender. Collagen was extracted with a subsequent addition of NaCl and acetic acid. Briefly, dry NaCl was added to the homogenate to a final concentration of 1 M, and the mixture was constantly stirred for 48 hours. The mixture was centrifuged (10 000 g, 30 min), the pellet containing collagen was solubilized in 0.5 M acetic acid, and the supernatant was salted-out by adding dry NaCl to a final concentration of 1 M. The mixture was stirred for 24 h. The resulting precipitate was collected by centrifugation (10 000 g, 30 min) and then dissolved in 0.5 M acetic acid. The samples of dissolved collagen were pooled. After dialysis against pure water, the samples were lyophilized in a freeze dryer and used to obtain collagen peptides. For this purpose, lyophilized collagen (1 g) was suspended in 20 mL of 0.2 M acetic acid and mixed with pepsin ( ~ 2500 units mg/protein, Sigma-Aldrich, St. Louis, MO, USA) at an enzyme:substrate ratio of 1: 100 (w/w). The mixture was stirred for 8 h at 37 °C. Hydrolysis was stopped by heating the sample at 95 °C for 10 min in a temperature-controlled water bath shaker. The samples were then centrifuged at 10 000 g for 15 min. Collagen peptides with a molecular weight of less than 10 kDa were isolated by ultrafiltration with a Pierce Protein Concentrator PES, 10K MWCO (Thermo Fisher Scientific, Waltham, MA, USA). The molecular weight of the obtained collagen peptides was assessed with SDS-polyacrylamide gel electrophoresis according to the Laemmli method (22). The obtained fraction of collagen peptides was further lyophilized. The quality control of peptide preparation is described in detail in the Supplemental material.

### Animals and experimental design

Thirty Wistar male rats (5 weeks old; an initial weight 90 ± 5 g) were used. All animal experiments complied with the principles of the Council of Europe Convention for the Protection of Vertebrate Animals Used for Experimental and Other Scientific Purposes. The study was approved by the Ethics Committee of Taras Shevchenko National University of Kyiv (protocol No. 1). The experiment was started one week after the acclimatization of the animals in the vivarium of Taras Shevchenko National University of Kyiv at constant temperature (22 ± 3 °C), humidity (60 ± 5%), and illumination (12 h light/12 h dark cycle). Standard food for rodents and water were provided *ad libitum*. On the eighth day, the animals were divided into the control (10 rats per group) and experimental (20 rats per group) group by simple randomization. The animals were kept in polypropylene cages (595 × 380 × 200 mm, floor area 1820 cm^2^); 5 rats in a cage. The control group received a basal rodent diet for the next 10 weeks. Rats in the experimental group received a high-calorie diet consisting of a standard meal (60%), lard (10%), eggs (10%), sugar (9%), peanuts (5%), dry milk (5%), and sunflower oil (1%) (23). All animals received food and water *ad libitum*. After four weeks, the experimental animals were randomly divided into two groups (10 rats per group). The rats in the first group continued to receive a high-calorie diet. The rats in the second group also received a high-calorie diet, but they were also intragastrically administered collagen peptides (1 g per kg of body weight) in 0.9% NaCl every other day for the next six weeks. The control group and the first experimental group were administered an equal volume of 0.9% NaCl. Food and water intake were measured daily at a fixed time. Body mass index (BMI) was calculated at the end of the experiment. At the end of the 10th week, the animals were not fed overnight and were then sacrificed. The serum was obtained by centrifugation (1000 g, 30 min) of blood samples preincubated at 37 °C for 30 min.

### Biochemical analysis

Glucose concentration was assessed with a Glucophot-II glucometer (Norma, Kyiv, Ukraine). Glycated hemoglobin was measured with an assay kit (Pliva-Lachema Diagnostika, Brno, Czech Republic). The insulin content in serum was measured by enzyme-linked immunosorbent assay according to a previously published protocol (24). The level of lipid peroxidation products was determined in reaction with a thiobarbituric acid reagent and expressed as thiobarbituric acid reactive substances (TBARS) (25). The level of Schiff bases and conjugated dienes was determined according to the method by Fletcher et al (26). The level of oxidatively modified proteins was assessed spectrophotometrically in the reaction with 2,4-dinitrophenylhydrazine (25). Superoxide dismutase activity (SOD) was determined spectrophotometrically based on the ability of the enzyme to inhibit the autoxidation of adrenaline (27). The protein concentration was measured according to the method by Bradford (28).

### Statistical analysis

Data are expressed as mean ± standard deviation (SD). The normality of distribution was assessed with a Kolmogorov-Smirnov test. The significance of differences between the groups was assessed with a one-way analysis of variance (ANOVA) with a Tukey *post hoc* test. The level of significance was *P* < 0.05. Statistical analysis was performed with STATISTICA, version 8.0 (StatSoft, Tulsa, OK, USA).

## RESULTS

Effect of collagen peptides on body mass index, body weight gain, and some nutritional parameters

To determine whether obesity was present in rats fed a high-calorie diet, BMI and body weight gain were assessed in all experimental groups. The BMI of the control rats was 0.62 ± 0.05 g/cm^2^ (Table 1), which was within the reference range for rats of this age (22). In rats fed high-calorie diet, BMI was 0.78 ± 0.05 g/cm^2^, which was 1.25 times higher than in control rats. The group treated with collagen peptides also experienced an increase in BMI, but it was significantly lower than that in obese rats.

The weight gain in the control group was 107.5%, among rats with HCD obesity it was 165.5%, and among rats administered collagen peptides it was significantly lower compared with the obese group (132%; *P* < 0.05) (Table 1).

To elucidate the possible mechanisms underlying the action of collagen peptides, we analyzed whether treatment with collagen fragments affected food consumption and water intake. The control group consumed an average of 23.5 ± 2.4 g of standard food per day. Obese rats consumed approximately 30.5 ± 2.4 g of high-calorie food per day. Rats administered collagen peptides consumed 20.5 ± 2.2 g of food per day, which was significantly lower (*P* < 0.05) compared with obese animals. No significant changes in water consumption were found between all experimental groups, with only a tendency toward a decreased water intake by obese animals.

### The effect of collagen peptides on parameters associated with insulin resistance

In order to evaluate the effect of collagen peptides on insulin resistance development, we assessed the levels of glucose, insulin, and glycated hemoglobin (HbA1c). The concentration of blood glucose decreased significantly (*P* < 0.05) in rats treated with collagen peptides (Table 2) compared with the value in obese rats. Insulin level increased 1.6 times in obese rats, but returned to the reference range in rats that received collagen peptides. HbA1c content substantially increased among obese rats – this value was 0.85 ± 0.06 μmol fructose/g hemoglobin compared with 0.38 ± 0.06 μmol fructose/g hemoglobin in control animals. The administration of collagen peptides significantly decreased HbA1c level (*P* < 0.05) compared with the level in obese rats.

### Effect of collagen peptides on markers of oxidative stress

To elucidate the mechanism of action of collagen peptides, their effect on oxidative status was determined. In obese rats, the level of lipid peroxidation products increased – the levels of conjugated dienes, TBARS, and Schiff bases exceeded the corresponding control values (Table 3). In addition, the level of oxidatively modified proteins significantly increased (4.5 times) compared with the control group. Compared with obese rats, rats administered collagen peptides had significantly decreased concentrations of conjugated dienes and Schiff bases. In contrast, TBARS level in rats administered collagen peptides was slightly higher than in obese animals (0.81 ± 0.004 nmol/mg protein vs 0.71 ± 0.003 nmol/mg protein). The activity of superoxide dismutase was significantly reduced (*P* < 0.05) in obese animals and significantly increased (*P* < 0.05) in rats treated with collagen peptides compared with the control group and obese rats.

## DISCUSSION

This study showed that an intragastric administration of jellyfish-derived collagen peptides for 6 weeks slowed down weight gain, reduced several parameters associated with insulin resistance, and reduced oxidative stress compared with the value in obese rats.

In recent years, there has been growing interest in the use of bioactive peptides as alternative agents for the treatment of metabolic disorders (29-31). Our previous study revealed the weight-lowering effect of collagen fragments prepared from fish scales (32).

In this study, animals that received a high-calorie diet and collagen peptides from *Dipulmaris antarctica* had a BMI similar to the control value. A similar effect of jellyfish collagen hydrolysate on body weight in mice fed a high-fat diet was previously demonstrated (33). However, collagen peptides may have a number of advantages over collagen hydrolysates from both a pharmacological and biotechnological point of view. Peptides are more stable than proteins and can be stored for a long time without a loss of biological activity. In addition, they exhibit a more pronounced activity; peptide preparations are well absorbed by various routes of administration and do not provoke an immune response.

Given that most peptide-containing products are consumed as functional foods or supplements, in the current study, collagen peptides were administered intragastrically to mimic the administration route in humans. Regardless of the etiology, obesity development is accompanied by impaired control of appetite, which leads to excessive food intake. The decrease in both BMI and body weight gain of rats treated with collagen peptides compared with obese rats may be explained by their reduced appetite. This may indicate the ability of collagen peptides to affect satiety. The exact mechanism of action of collagen peptides was not established. However, given that we used a mixture of peptides, it can be assumed that the anti-obesity effect of jellyfish collagen peptides is complex, that it is realized at different levels, and that it involves various mechanisms. Enzymatic hydrolysis of jellyfish collagen results in the formation of many peptides, some of which are bioactive. As collagen peptides are possibly structurally similar to several gut hormones and neuropeptides involved in the regulation of energy homeostasis, they may influence food intake and satiety in rats by mimicking the action of gut peptide hormones. Another study also found that peptides isolated from shrimp influenced the release of cholecystokinin by STC-1 cells, leading to appetite suppression (34). Similarly, milk protein hydrolysates were shown to bind to serotonin receptors, creating an appetite-suppression effect similar to that of physiological ligands (35).

Many studies show obesity to be an important trigger of type-2 diabetes mellitus, the progression of which is characterized by impaired glucose homeostasis (36). In our experiments, an increased level of HbA1c in rats fed a high-calorie diet indicates an increase in glucose concentration over a long period. The simultaneous increase in glucose and insulin concentrations in obese animals may predict the development of insulin resistance. Additionally, hyperinsulinemia is a compensatory reaction of pancreatic beta cells to a decrease in tissue sensitivity to the action of insulin. The ability of collagen fragments derived from various sources to modulate glucose and lipid metabolism was previously confirmed (37,38). Considering this fact, we examined whether collagen peptides have the same effect on the parameters associated with insulin resistance. The obtained data indicate a decrease in the level of glucose, insulin, and HbA1c in obese rats treated with collagen peptides. Since an increased content of abdominal fat (confirmed by a high BMI) and, accordingly, an increased level of non-esterified fatty acids and inflammatory markers lead to decreased insulin sensitivity, the recovery of parameters associated with insulin resistance may be explained by the effect of collagen peptides on body weight and body fat content.

Another trigger for the development of obesity-related disorders is systemic oxidative stress (39). Uncontrolled formation of reactive oxygen species (ROS) can be deleterious by itself since it causes oxidative damage to proteins, lipids, and nucleic acids and leads to the formation of aggressive secondary by-products. ROS action can cause oxidative modification of enzymes and changes in their function, damage pancreatic cells (40), and impair the insulin responsiveness of muscle and liver cells. In addition, both ROS and lipid peroxidation products can disturb redox-dependent cellular homeostasis and signal-transduction pathways, leading to apoptosis, increased inflammation, adipokine imbalance, and even changes in neurotransmitter activity. All of these factors, individually or in combination, are involved in the induction of insulin resistance in obesity. Moreover, our study found an accumulation of oxidatively modified proteins in obese rats. This may further indicate the intensity and duration of oxidative stress as oxidatively modified proteins are an early criterion of tissue damage by free radicals. If the structure of cellular proteins is modified by free radicals, their function is decreased or completely abolished. This may cause an accumulation of protein aggregates – factors provoking the development of complications associated with obesity. In addition, proteins that undergo oxidative modification may stimulate the production of new antibodies, thus provoking an immune or autoimmune response. Oxidative stress often occurs as a result of a decreased activity of antioxidant defense system due to the depletion of non-enzymatic antioxidants with low-molecular-weight. It may also occur due to a decreased level and activity of antioxidant enzymes. In our experiment, the presence of oxidative stress in obese rats was confirmed by a decreased activity of superoxide dismutase. A previous study also found a decreased activity of antioxidant enzymes and a depletion of antioxidants in overweight and obese animals (41).

In our experiments, collagen peptides prevented the development of oxidative stress in rats fed a high-calorie diet. They restored the capacity of the antioxidant system, as shown by the normalized level of oxidative-modified proteins and an increased activity of superoxide dismutase. This may be the result of a direct action of peptides as free-radical scavengers. Previous *in vitro* and *in vivo* studies confirmed the ability of peptides to reduce the levels of superoxide radicals, hydroxyl radicals, and to chelate prooxidative transition metals (42,43). In addition, peptides may be involved in the regulation of several antioxidant enzyme genes. Given that oxidative stress is involved in the development of diabetic complications, achieving oxidative homeostasis in rats treated with collagen peptides may additionally reduce the damage to pancreatic cells.

The limitation of this study is the lack of a control group of animals without obesity administered with collagen peptides. Our study also did not assess the levels of pro-inflammatory cytokines, as well as the levels of key gut and adipose tissue hormones, which is a step needed to comprehensively assess the anti-obesity effect of collagen peptides.

To our knowledge, this is the first report to evaluate the effect of collagen peptides from the jellyfish *Diplulmaris antarctica*. Due to its abundance and high proliferative potential, this jellyfish can be considered a sustainable source of collagen and its derivatives. Given the conservatism of the collagen structure, jellyfish from other regions can also be used to obtain collagen peptides, which may contribute to curbing their uncontrolled spread in the oceans. Our results indicate that jellyfish collagen peptides can be used for the prevention and treatment of obesity caused by a high-calorie diet, as well as of pathologies associated with increased oxidative stress. Further studies are needed to elucidate the mechanisms of the anti-obesity effect of jellyfish collagen peptides.

**Table:** **Тable 1.** The effect of collagen peptides on body mass index, weight gain, and some nutritional parameters in obese rats*

Parameters	Rats		
	control	obese	administered collagen peptides
Body mass index (g/cm^2^)	0.62 ± 0.05	0.78 ± 0.05^†^	0.67 ± 0.05^‡^
Body weight gain, %	107.5 ± 4.7	165.5 ± 8.0^†^	132.0 ± 6.5^†‡^
Food intake (g/day)	23.5 ± 2.4	30.5 ± 2.4^†^	20.5 ± 2.2^‡^
Water intake (mL/day)	35.6 ± 4.4	28.6 ± 3.6	32.5 ± 3.7

*Data are expressed as mean ± standard deviation (n = 10).

†*P* < 0.05 significantly different from the control group.

‡*P* < 0.05 significantly different from the group of obese rats.

**Table:** **Тable 2.** The effect of collagen peptides on fasting blood glucose concentration, insulin level, and glycated hemoglobin (HbA1c) level in three groups of rats*

	Rats		
Parameters	control	obese	administered collagen peptides
Fasting blood glucose, mmol/L	5.60 ± 0.50	7.66 ± 0.50^†^	6.35 ± 0.50^‡^
HbA1c, μmol fructose/g hemoglobin	0.38 ± 0.04	0.85 ± 0.06^†^	0.60 ± 0.06^†‡^
Insulin, relative units	0.13 ± 0.006	0.21 ± 0.010^†^	0.14 ± 0.006^‡^

*Data are expressed as mean ± standard deviation (n = 10).

†*P* < 0.05 significantly different from the control.

‡*P* < 0.05 significantly different from obese rats.

**Table:** **Тable 3.** The effect of collagen peptides on the levels of lipid peroxidation products, oxidatively modified proteins, and activity of superoxide dismutase in the blood of obese rats

Parameters	Rats		
	control	obese	administered collagen peptides
Conjugated dienes, nmol/mg protein	0.029 ± 0.0009	0.046 ± 0.002^†^	0.031 ± 0.0007^‡^
Thiobarbituric acid reactive substances, nmol/mg protein	0.03 ± 0.001	0.71 ± 0.003^†^	0.81 ± 0.004^†‡^
Schiff bases, relative units/mg protein	45.3 ± 2.45	173.7 ± 8.15^†^	56.2 ± 4.23^†‡^
Superoxide dismutase activity, U/mg protein/min	3.44 ± 0.38	2.05 ± 0.41	4.75 ± 0.42^†‡^
Oxidative modified proteins, nmol/mg protein	0.16 ± 0.09	0.73 ± 0.05^†^	0.27 ± 0.02^†‡^

*Data are expressed as mean ± standard deviation (n = 10).

†*P* < 0.05 significantly different from the control group.

‡*P* < 0.05 significantly different from obese rats.
